# Associations Between Physical, Cognitive, and Mental Health Domains of Post-Intensive Care Syndrome and Quality of Life: A Longitudinal Multicenter Cohort Study

**DOI:** 10.1097/CCM.0000000000006461

**Published:** 2024-10-24

**Authors:** Bram Tilburgs, Koen S. Simons, Stijn Corsten, Brigitte Westerhof, Thijs C. D. Rettig, Esther Ewalds, Marieke Zegers, Mark van den Boogaard

**Affiliations:** 1 Department of Intensive Care Medicine, Radboud University Medical Center, Nijmegen, The Netherlands.; 2 Department of Intensive Care Medicine, Jeroen Bosch Hospital, ’s-Hertogenbosch, The Netherlands.; 3 Department of Intensive Care Medicine, Canisius Wilhelmina Hospital, Nijmegen, The Netherlands.; 4 Department of Intensive Care Medicine, Rijnstate Hospital, Arnhem, The Netherlands.; 5 Department of Anesthesiology, Intensive Care and Pain Medicine, Amphia Hospital, Breda, The Netherlands.; 6 Department of Intensive Care Medicine, Bernhoven Hospital, Uden, The Netherlands.

**Keywords:** cognitive health, intensive care, mental health, physical health, post-intensive care syndrome, quality of life

## Abstract

**OBJECTIVES:**

To explore associations between the physical, cognitive, and mental post-intensive care syndrome (PICS) health domains with changes in health-related quality of life (HRQoL) following ICU admission.

**DESIGN:**

A longitudinal prospective multicenter cohort study.

**SETTING/PATIENTS:**

Patients (*n* = 4092) from seven Dutch ICUs.

**INTERVENTIONS:**

None.

**MEASUREMENTS AND MAIN RESULTS:**

At ICU admission, 3 and 12 months post-ICU, patients completed validated questionnaires regarding physical health problems, cognitive health problems, mental health problems, and HRQoL. Composite scores were created for the physical health domain (physical problems and fatigue) and mental health domain (anxiety, depression, and post-traumatic stress disorder). Adjusted multivariable linear regression analyses were performed, including covariables (e.g., patient characteristics, disease severity, pre-ICU HRQoL, etc.) to explore associations between the physical, cognitive, and mental health domains of PICS and changes in HRQoL at 3 and 12 months post-ICU. At 3 months (*n* = 3368), physical health problems (β = –0.04 [95% CI, –0.06 to 0.02]; *p* < 0.001), cognitive health problems (β = –0.05 [95% CI, –0.09 to –0.02]; *p* < 0.001), and mental health problems (β = –0.08 [95% CI, –0.10 to –0.05]; *p* < 0.001) were negatively associated with changes in HRQoL. Also, at 12 months (*n* = 2950), physical health problems (β = –0.06 [95% CI, –0.08 to –0.03]; *p* < 0.001), cognitive health problems (β = –0.04 [95% CI, –0.08 to –0.01]; *p* < 0.015), and mental health problems (β = –0.06 [95% CI, –0.08 to –0.03]; *p* < 0.001) were negatively associated with changes in HRQoL.

**CONCLUSIONS:**

PICS symptoms in the physical, cognitive, and mental domains are all negatively associated with changes in HRQoL at 3 and 12 months post-ICU. At 3 months, PICS symptoms in the mental domain seem to have the largest negative associations. At 12 months, the associations of PICS in the mental and physical domains are the same. This implies that daily ICU care and follow-up care should focus on preventing and mitigating health problems across all three PICS domains to prevent a decrease in HRQoL.

**KEY POINTS**

**Question**: To explore associations between post-intensive care syndrome (PICS) and changes in health-related quality of life (HRQoL) at 3 and 12 months post-ICU.

**Findings**: This longitudinal prospective multicenter cohort study showed that PICS symptoms in the physical, cognitive, and mental domain are all significantly negatively associated with changes in long-term HRQoL. PICS symptoms in the mental domain seem to have the largest impact on changes in long-term HRQoL, followed by the physical and cognitive domains.

**Meaning**: Because of the negative impact of PICS symptoms on changes in HRQoL, daily ICU care, and follow-up care focus should on preventing and mitigating PICS in all domains.

Worldwide millions of people are admitted to an ICU each year. Due to progress in ICU treatment and technological innovations, ICU mortality has decreased over the last decades, resulting in more survivors (1–3). Even though patients’ survival is one of the main goals of ICU treatment, the importance of health-related quality of life (HRQoL) post-ICU has gained interest as an important outcome (4).

An ICU admission has a large impact on patients. During their admission, patients experience, immobilization, pain, loss of autonomy, and anxiety and often have limited understanding of their situation (5–7). On the long term, ICU patients frequently experience physical, cognitive, and mental problems. When such problems arise or worsen after critical illness and persist beyond hospitalization, this is defined as the post-intensive care syndrome (PICS) (8). Around 50% of ICU patients experience PICS 1-year post-ICU (9). ICU survivors indicate that physical, cognitive, and mental problems have a major impact on their lives (10). Many continue in need of care and face changes in employment (11). Even though HRQoL improves over time, ICU survivors’ HRQoL remains lower than general population levels (12).

Although the relationship between PICS and HRQoL appears logical, this has not been well demonstrated. In previous studies with limited numbers of patients, pre-ICU HRQoL was not included, and most patients were not followed for more than half a year (13, 14). These are omissions since pre-ICU HRQoL appears to be a significant predictor for long-term HRQoL, and many patients experience PICS problems up to 1-year post-ICU (9, 10). It is important to study which PICS domains at which point in time are associated with changes in HRQoL. This supports the assertation that preventing PICS enhances HRQoL for ICU patients and emphasizes the importance of providing ICU care that minimizes the long-term impact admission. We, therefore, aimed to explore associations between the physical, cognitive, and mental PICS with changes in HRQoL between baseline and 3 months and between baseline and 12 months post-ICU.

## METHODS

### Design and Setting

This is a substudy of the Monitoring cOnsequeNces of InTensive care fOR Intensive Care patients (MONITOR-IC), an ongoing prospective cohort study in which long-term outcomes of ICU patients are collected in seven Dutch hospitals (www.clinicaltrials.gov identifier NCT03246334) (9, 15). The MONITOR-IC is conducted according to the declaration of Helsinki and approved by the research ethics committee of the region Arnhem-Nijmegen (Monitoring cOnsequeNces of InTensive care fOR Intensive Care patients, August 23, 2016, nr2016-2724). The study is reported according to the Strengthening the Reporting of Observational Studies in Epidemiology (STROBE) guidelines (**Supplemental file**, http://links.lww.com/CCM/H602).

### Study Population

ICU patients admitted between March 2017 and March 2020 who completed the baseline questionnaire were eligible (9, 15). Included patients were 16 years old or older, admitted greater than 12 hours to the ICU and provided informed consent. We choose greater than or equal to 12 hours as a criteria because this usually means that patients stay in the ICU for at least one night and it is not ruled out that even a single night can have long-term consequences. Patients were excluded when they had a life expectancy of less than or equal to 48 hours, received palliative care, were admitted for a donor procedure, were unable to understand Dutch, or were not able (or their legal representative) to complete the questionnaire (9, 15).

### Data Collection

Patients were approached to complete the questionnaire at ICU admission (baseline) and after 3 and 12 months. At baseline, respondents were asked to reflect their health situation before ICU admission. Reminders were sent after 4 and 6 weeks (9, 15). Elective surgical patients were recruited at the outpatient clinic before ICU admission. Nonelective patients were recruited at the ICU. If a nonelective patient was unable to give consent or complete the questionnaire, the legal representative was asked (9, 15). Demographics, admission characteristics, and data related to patients’ health status, including the PICS domains, were collected using questionnaires and medical records. Education was assessed at three levels: 1) low: primary and lower vocational education; 2) medium: lower secondary and intermediate vocational education; and 3) high: higher secondary, professional education, and university. Composite scores were calculated for the PICS domains (physical, cognitive, and mental, see *Below*). This method, based on previous studies, was chosen to limit the number of PICS-related independent variables and facilitate the interpretation of data (8, 9, 16).

### Physical Domain

PICS’ physical domain consists of: new or worsened physical problems and fatigue. New or worsened physical problems (e.g., shortness of breath) post-ICU were assessed with a 30-item questionnaire with a 4-point Likert scale: 0 (no problems), 1 (few problems), 2 (moderate problems), and 3 (severe problems). This questionnaire was developed by the Netherlands Institute for Health Services Research, the Radboudumc ICU research team, and ICU patient’s representatives (15). Scores on physical problems were dichotomized into 0 (patients experienced no [0] or few physical problems [1]) or 1 (patients experienced moderate [2] or severe problems [3]) (9).

Fatigue was assessed with the eight-item Checklist Individual Strength (CIS). Respondents rated statements (e.g., I feel tired) on a 7-point Likert scale from 0 (not correct) to 7 (correct). The total scores range from 8 to 56 using the validated cutoff score of greater than or equal to 27 indicating fatigue (17). Scores were dichotomized into 0 (score < 27, no fatigue) or 1 (score 27, ≥ fatigue) (9). Composite scores were created for PICS’ physical domain. When patients scored “1” on physical problems (dichotomized) or scored “1’ on fatigue (dichotomized), the composite score on PICS” physical domain was scored 1 (opposed to 0).

### Cognitive Domain

Cognitive problems were assessed with the 14-item Cognitive Failure Questionnaire (CFQ) (18). Respondents rate statements (e.g., having trouble with decision-making) on a 5-point Likert scale from 0 (never) to 4 (very often). Overall scores are factored to a range from 0 to 100 (19). When patients scored greater than 43 (the validated cutoff score), the PICS’ cognitive domain was scored 1 (opposed to 0) (18).

### Mental Domain

PICS’ mental domain consists of symptoms of anxiety, depression, and post-traumatic stress disorder (PTSD). Anxiety and depression were assessed with the 14-item Hospital Anxiety and Depression Scale (HADS) (20). The HADS contains of two subscales with seven items for anxiety and seven items for depression. Respondents rate statements (e.g., I feel tense) on a 4-point Likert scale from 0 (not at all) to 3 (very often) with a possible score from 0 to 21. A score of greater than or equal to 8 (the validated cutoff score) on a subscale indicates symptoms of anxiety or depression (20). Scores for anxiety or depression were dichotomized into 0 (score < 8, no anxiety or depression) or 1 (score ≥ 8, anxiety or depression) (9).

ICU admission related PTSD was assessed with the six-item version of the Impact of Event Scale (IES-6) (21). Respondents rated statements (e.g., I tried not to think about it) on a 5-point Likert scale from 0 (not at all) to 4 (extremely) with a possible overall mean score ranging from 0 to 4. A mean score of greater than or equal to 1.75 (the validated cutoff score) was used to indicate symptoms of PTSD (21). Scores for PTSD were dichotomized into 0 (score < 1.75, no PTSD) or 1 (score ≥ 1.75, PTSD) (9). Composite scores were created for PICS’ mental domain. When patients scored 1 (dichotomized) on anxiety, depression, or PTSD, the composite score on PICS’ mental domain was score 1 (opposed to 0).

### Health-Related Quality of Life

HRQoL was assessed using the European Quality of Life 5D Five Level Version (EQ5D-5L). The EQ5D-5L defines health on five dimensions (mobility, self-care, usual activities, pain/discomfort, and anxiety/depression) on five levels (no problem (1) to extreme problems (5)) (22). Scores were converted to a Dutch index value from –0.446 to 1 (23). On this scale, scores range from 1 (fully functional quality of life) to 0 (death) but also allow for negative scores. A value of less than 0 represents a health state worse than death (24). Changes between baseline HRQoL and 3 months and between baseline and 12 months were calculated by subtracting baseline HRQoL from HRQoL at 3 and 12 months and are, as such, presented as delta scores.

### Statistical Analyses

Patients who completed the baseline questionnaire, the 3 and/or 12 months follow-up were included in the analyses. Because of possible participation bias, differences between responders and nonresponders at 3 and 12 months were assessed using chi-square tests, Mann-Whitney *U* tests, or independent-sample *t* tests. Mean and sds were used to describe normally distributed data. For not normally distributed data, median and interquartile range (IQR) were used. Numbers and percentages were used to describe categorical data.

In accordance with the relevant manuals, missing values on the CIS and HADS were imputed using the half-rule (15). IES-6 scores were replaced by their individual mean when 75% of the items were completed (15). Missing values from the new or worsened physical complaints questionnaire were replaced by 0 (no complaints). Regarding the CFQ, when only one item was missing and the patients total score was above the cutoff value of 43, the missing value was imputed with 4 (very often). When the patients total score was less than 39 with only one missing, we also imputed the missing with 4 (very often). Since the cutoff score of the CFQ is 43, the dichotomized score did not change.

To explore associations between PICS domains and changes in HRQoL, unadjusted and adjusted univariable and multivariable linear regression analyses were carried out for the 3- and 12-month data (25, 26). In the crude analyses, physical, cognitive, and mental PICS domains were included as independent variables and changes in HRQoL as the dependent variable. Age, gender, education, length of ICU stay, disease severity, type of admission, and household composition served as covariables in the adjusted analyses and were selected based on previous research (10, 27–31). Multicollinearity concerning all independent variables (including the physical, cognitive, and mental PICS domains composite scores) was studied using the variance influence factor (VIF) and tolerance. VIF and tolerance values for all independent variables were less than 10 and greater than 0.1. It was therefore concluded that collinearity was of no concern (26). Because a large number of patients in the study were elective surgical patients and may be different compared with nonelective patients, they were also analyzed separately. All statistical analyses were performed using IBM SPSS Statistics for Windows, Version 27, released 2020 (IBM Corp., Armonk, NY) and *p* values of less than 0.05 were considered to indicate statistical significance. To report this study, the STROBE checklist for cohort studies was used (32).

## RESULTS

### Characteristics of the Study Population

**Figure 1.**
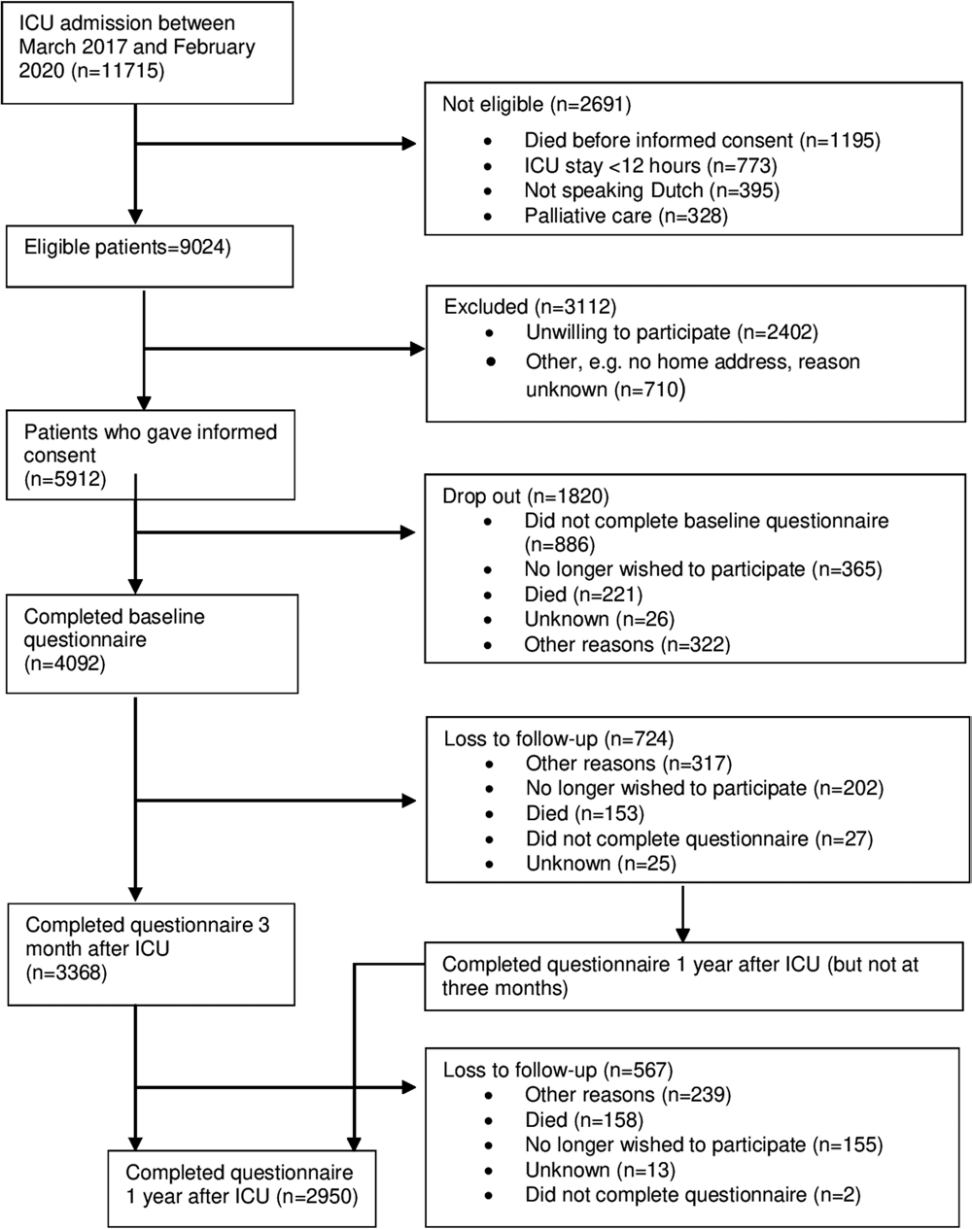
Flowchart of study inclusions.

Of the 11,715 ICU patients admitted between March 2017 and March 2020, 9,024 patients were eligible. Five thousand nine hundred twelve patients gave informed consent. Of these 5912 patients, 4092 (69%) completed the baseline questionnaire. At 3 months, 3368 (57%) patients completed the questionnaire and 724 patients (12%) were lost to follow-up. At 12 months, 2950 of the 5912 patients (50%) completed the questionnaire, 567 (9%) patients were lost to follow-up, and 149 patients (3%) who did not complete the 3 months questionnaire did complete the questionnaire at 12 months (**Fig. 1**).

A total of 47% of the patients were between 65 and 79 years, and 65% were male and 54% were admitted to an ICU for a planned surgical reason. The mean Acute Physiology and Chronic Health Evaluation IV score was 55 (± 21.9) and median length of ICU stay was 1.1 days (IQR, 0.9–2.8 d). Before ICU admission (baseline), the mean HRQoL was 0.70 (± 0.3), 64% was fatigued, 6% had cognitive problems, 27% had symptoms of anxiety, and 26% had symptoms of depression (**Table 1**, Respondent’s baseline characteristics including missing (**Supplemental table 1**, http://links.lww.com/CCM/H602). Baseline characteristics of elective surgical patients are included in **Supplemental table 2** (http://links.lww.com/CCM/H602).

**TABLE 1.** Respondent’s Baseline Characteristics

Patient Characteristics	Respondents (*n* = 4092)
Age, yr, *n* (%)	
16–39	253 (6.2)
40–64	1574 (38.5)
65–79	1904 (46.5)
> 80	269 (6.6)
Sex, *n* (%)	
Male	2640 (64.5)
Female	1450 (35.4)
Household composition, *n* (%)	
Living alone	700 (17.1)
Living with someone else	3338 (81.6)
Living in nursing home	54 (1.3)
Level of education, *n* (%)	
Low	1366 (33.4)
Medium	1715 (41.9)
High	948 (23.2)
Admission type, *n* (%)	
Medical	1327 (32.4)
Planned surgical	471 (11.5)
Acute surgical	2197 (53.7)
Admission reason, *n* (%)	
Cardiovascular	2397 (59)
Respiratory	573 (14)
Neurologic	366 (9)
Gastrointestinal	285 (7)
Trauma	198 (5)
Other	271 (6)
Mean Acute Physiology and Chronic Health Evaluation IV score (sd)	54.9 (21.9)
Median length of ICU stay (interquartile range), d	1.1 (0.9–2.8)
> 24 hr of mechanical ventilation (%)	
No	1398 (34.2)
Yes	2602 (63.6)
Mean quality of life; European Quality of Life 5D Five Level Version (sd)	0.70 (0.3)
Fatigue; Checklist Individual Strength, *n* (%)	
No fatigue	1425 (34.8)
Fatigue	2626 (64.2)
Cognition; Cognitive Failure Questionnaire, *n* (%)	
No cognitive problems	3592 (87.8)
Cognitive problems	251 (6.1)
Anxiety; Hospital Anxiety and Depression Scale-Anxiety, *n* (%)	
No symptoms of anxiety	2978 (72.8)
Symptoms of anxiety	1092 (26.7)
Depression; Hospital Anxiety and Depression Scale-Depression, *n* (%)	
No symptoms of depression	3023 (73.9)
Symptoms of depression	1049 (25.6)

Missing values are presented in the Supplemental table 9 (http://links.lww.com/CCM/H602).

Responders at 3 and 12 months differed from nonresponders. Nonresponders were more often female, older than 80 years, low educated, admitted for a medical reason, longer admitted to the ICU, and more fatigued. At baseline, they experienced a lower HRQoL, more cognitive problems, and more symptoms of anxiety and depression (**Supplemental table 3**, http://links.lww.com/CCM/H602).

### Frequency of Post-ICU Health Problems

At 3 months, 75% of former ICU patients reported PICS in any of the three domains: 72% reported PICS in the physical domain, 11% reported PICS in the cognitive domain, and 33% reported PICS in the mental domain.

At 12 months, 71% of former ICU patients reported PICS in any of the three domains: 68% reported PICS in the physical domain, 12% reported PICS in the cognitive domain, and 32% reported PICS in the mental domain.

### PICS Domains and Associations With Changes in HRQoL Between Baseline and 3 Months

**Figure 2.**
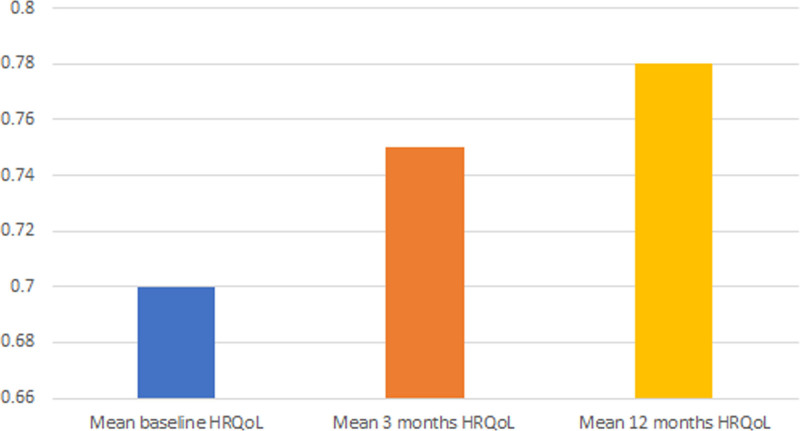
Mean baseline, 3 mo, and 12 mo health-related quality of life (HRQoL).

**Figure 3.**
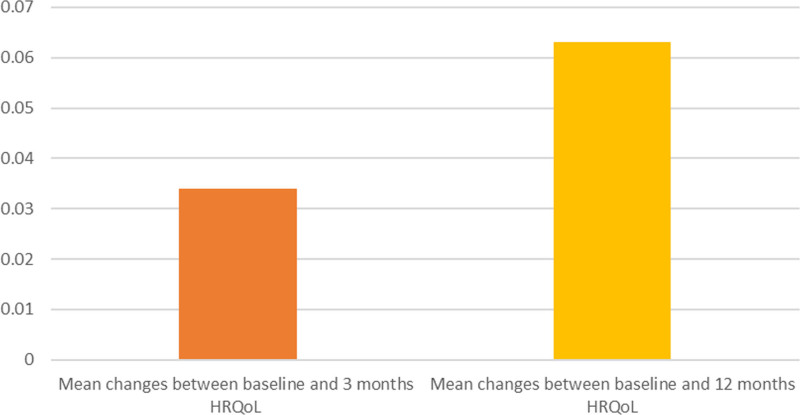
Mean changes between baseline and 3 mo and baseline and 12 mo health-related quality of life (HRQoL).

At 3 months, patients reported a mean HRQoL of 0.75 (sd, 0.24) and a mean difference between baseline and 3 months HRQoL of 0.034 (sd, 0.29) (**Figs. 2** and **3**). In the unadjusted analyses, all PICS domains were significantly and negatively associated with changes in HRQoL. PICS in the mental domain had the highest negative unadjusted beta (β = –0.07 [–0.09 to –0.05]; *p* ≤ 0.001). This means that when there is a positive score on the mental domain of PICS, the difference between baseline and 3 months HRQoL score decreases with 0.07 (**Supplemental table 4**, http://links.lww.com/CCM/H602). In the adjusted multivariate analyses, all PICS domains were significantly, negatively associated with changes in HRQoL. PICS in the mental domain had highest negative adjusted beta (β = –0.08 [–0.10 to –0.05]; *p* ≤ 0.001) (**Table 2**). To summarize, the unadjusted and adjusted univariable analyses all show the same pattern, namely that all PICS domains are significantly and negatively associated with changes in HRQoL. PICS in the mental domain is most negatively associated with changes in HRQoL (**Supplemental tables 5–10**, http://links.lww.com/CCM/H602).

**TABLE 2.** Adjusted Associations of Physical, Cognitive, and Mental Post-Intensive Care Syndrome Domains With Differences Between Baseline and 3 Months Post-ICU Health-Related Quality of Life

Patient Characteristics	β (95% CI)	se	Standardized Coefficients β	*p*	Tolerance	Variance Influence Factor
Constant	0.09 (0.02–0.16)	0.03		0.01		
Physical PICS	–0.04 (–0.06 to –0.02)	0.01	–0.06	< 0.001	0.84	1.2
Cognitive PICS	–0.05 (–0.09 to –0.02)	0.02	–0.06	< 0.001	0.90	1.1
Mental PICS	–0.08 (–0.10 to –0.05)	0.01	–0.13	< 0.001	0.79	1.3
Gender	0.03 (0.01–0.05)	0.01	0.06	< 0.001	0.94	1.1
Age						
18–39 (reference)						
40–64	–0.03 (–0.07 to 0.02)	0.02	–0.05	0.22	0.19	5.2
65–79	–0.02 (–0.07 to 0.03)	0.02	–0.04	0.40	0.18	5.6
> 80	–0.02 (–0.08 to 0.04)	0.03	–0.01	0.60	0.47	2.1
Education						
Low (reference)						
Medium	–0.01 (–0.03 to 0.02)	0.01	–0.01	0.64	0.72	1.4
High	–0.04 (–0.07 to –0.01	0.01	–0.06	< 0.001	0.71	1.4
Household composition						
Alone (reference)						
With someone else	0.01 (0.00–0.03)	0.01	0.02	0.14	0.92	1.1
Nursing home	–0.12 (–0.19 to –0.06)	0.03	–0.05	< 0.001	0.94	1.1
Admission type						
Medical (reference)						
Acute surgical	–0.04 (–0.08 to –0.01)	0.02	–0.05	0.01	0.82	1.2
Planned surgical	–0.01 (–0.03 to 0.02)	0.01	–0.01	0.64	0.69	1.4
Acute Physiology and Chronic Health Evaluation IV score	0.00 (0.00–0.00)	0.00	0.00	0.91	0.75	1.3
Length of ICU stay	0.00 (0.00–0.00)	0.00	–0.05	0.02	0.81	1.2

PICS = post-intensive care syndrome.

Planned surgical patients reported a mean HRQoL of 0.79 (sd, 0.19) at 3 months and a mean difference between baseline and 3 months HRQoL of 0.06 (sd, 0.23). In the adjusted analysis, mental PICS (β = –0.061 [–0.09 to –0.04]; *p* ≤ 0.001) and physical PICS (β = –0.033 [–0.06 to –0.01]; *p* = 0.005) were significantly associated with changes in HRQoL (**Supplemental tables 11** and **12**, http://links.lww.com/CCM/H602).

### PICS Domains and Associations With Changes in HRQoL Between Baseline and 12 Months

At 12 months, patients reported a mean HRQoL of 0.78 (sd, 0.21) and a mean difference in HRQoL of 0.063 (sd, 0.027) between baseline and 12 months (Figs. 2 and 3). In the unadjusted analyses, all PICS domains were significantly and negatively associated with changes in HRQoL. PICS in the mental (β = –0.05 [–0.09 to –0.02]; *p* ≤ 0.001) and physical domain (β = –0.05 [–0.08 to –0.03]; *p* ≤ 0.001) had the highest negative unadjusted beta (**Supplemental table 13**, http://links.lww.com/CCM/H602) In the adjusted analyses, PICS in the mental (β = –0.06 [–0.08 to –0.03]; *p* ≤ 0.001) and physical (β = –0.06 [–0.08 to –0.03]; *p* ≤ 0.001) domains had the highest negative adjusted betas (**Table 3**). To summarize, the adjusted and adjusted univariable analyses show the same pattern, namely that all PICS domains are significantly and negatively associated with changes in HRQoL. PICS in the mental and physical PICS are most negatively associated with changes in HRQoL (**Supplemental tables 14–19**, http://links.lww.com/CCM/H602).

**TABLE 3.** Adjusted Associations of Physical, Cognitive, and Mental Post-Intensive Care Syndrome Domains With Health-Related Quality of Life 12 Months Post-ICU

Patient Characteristics	β (95% CI)	se	Standardized Coefficients β	*p*	Tolerance	Variance Influence Factor
Constant	0.08 (0.00–0.15)	0.038		0.04		
Physical PICS	–0.06 (–0.08 to –0.03)	0.013	–0.10	< 0.001	0.79	1.3
Cognitive PICS	–0.04 (–0.08 to –0.01)	0.017	–0.05	0.015	0.85	1.2
Mental PICS	–0.06 (–0.08 to 0.03)	0.013	–0.10	< 0.001	0.75	1.3
Gender	0.05 (0.02–0.07)	0.012	0.08	< 0.001	0.93	1.1
Age						
18–39 (reference)						
40–64	–0.03 (–0.08 to 0.02)	0.025	–0.05	0.30	0.19	5.4
65–79	–0.04 (–0.09 to 0.02)	0.026	–0.07	0.17	0.17	5.9
> 80	–0.04 (–0.10 to 0.03)	0.033	–0.03	0.25	0.46	2.2
Education						
Low (reference)						
Medium	0.01 (–0.03 to 0.02)	0.013	–0.02	0.49	0.69	1.4
High	–0.03 (–0.06 to –0.00)	0.014	–0.05	0.03	0.68	1.5
Household composition						
Alone (reference)						
With someone else	0.04 (0.01–0.07)	0.015	0.06	0.01	0.93	1.1
Nursing home	–0.07 (–0.19 to 0.06)	0.064	–0.02	0.31	0.95	1.1
Admission type						
Medical (reference)						
Acute surgical	–0.03 (–0.06 to 0.01)	0.018	–0.03	0.11	0.81	1.2
Planned surgical	0.01 (–0.02 to 0.03)	0.013	0.01	0.69	0.68	1.5
Acute Physiology and Chronic Health Evaluation IV score	0.00 (–0.00 to 0.00)	0.01	–0.01	0.69	0.76	1.3
Length of ICU stay	0.00 (–0.00 to 0.00)	0.00	–0.00	0.89	0.81	1.2

PICS = post-intensive care syndrome.

*R* = 0.69; *R*^2^ = 0.47; *p* ≤ 0.001.

Planned surgical patients reported a mean HRQoL of 0.82 (sd, 0.18) at 12 months and a mean difference between baseline and 12 months HRQoL of 0.08 (sd, 0.22). In the adjusted analysis, physical PICS (β = –0.062 [–0.09 to –0.04]; *p* ≤ 0.001) and mental PICS (β = –0.045 [–0.07 to –0.02]; *p* ≤ 0.002) were significantly associated with differences in HRQoL (**Supplemental tables 20** and **21**, http://links.lww.com/CCM/H602).

## DISCUSSION

This large multicenter prospective cohort study demonstrated that at 3 months post-ICU, PICS in the mental domain showed the most negative association with changes between baseline and 3 months HRQoL. At 12 months, PICS in the mental and physical domain showed equal negative associations. PICS in the cognitive domain is also negatively associated at 3 and 12 months post-ICU. The same picture emerges with selective surgical patients, although associations are lower and PICS in the cognitive domain is no longer significant. The number of selective surgical patients in this study may explain the increase in HRQoL at 3 and 12 months compared with baseline because planned surgery is usually performed to solve an existing health problem.

Previous research showed that physical, cognitive, and mental problems were negatively associated HRQoL. However, this was a single timepoint study, on average 6 months after ICU admission. In addition, only ICU patients who visited a follow-up clinic were approached (13). Another cohort study found negative associations between all PICS domains and HRQoL (14). However, this research involved a limited number of patients (*n* = 121) and only collected data from follow-up clinic patients with a range of 35–1713 days after admission (14). None of these studies used changes between baseline and 3 or 12 months HRQOL in there analyses. Since baseline HRQoL is known to be associated with long-term HRQoL (9), this could have caused an over estimation of their results. In addition, because of the large number of patients, the current study provided more robust evidence regarding the association between PICS and HRQoL.

Former research showed that the minimal clinical important difference for HRQoL, measured with the EQ5D, varies between 0.03and 0.52. The results we found are therefore not only statistically but also clinically important (33–35). New or aggravated symptoms of PICS significantly impact the decrease of HRQoL. Therefore, preventing or reducing these symptoms directly affects the experienced quality of life of former ICU patients.

The domains addressed in the EQ5D (mobility, self-care, usual activities, pain/discomfort, and anxiety/depression) provide an additional explanation concerning the magnitude of the significant associations between PICS and HRQoL. PICS’ physical problems likely results to issues with mobility, self-care, daily activities, and pain/discomfort (10). PICS’ problems of anxiety, depression, and PTSD are likely to result in issues with anxiety/depression of the EQ5D (36). Symptoms of PICS in the mental domain can also influence, discomfort, and daily activities because of factors such as stress, a deficiency in self-efficacy, coping abilities, or motivation (36, 37). However, problems in the PICS’ cognitive domain have, at first glance, a weaker relation with the domains of the EQ5D, which may explain the lesser association with HRQoL at 12 months.

Our study results with associations do not imply a causal relationship between PICS and changes in HRQoL. Therefore, caution should be exercised when translating our findings into practice. However, this does not lessen the belief that efforts should be directed toward the prevention or mitigation of PICS. Initiatives to prevent or limit PICS should address physical, mental, and cognitive symptoms comprehensively. Strategies encompassing early mobilization, early awakening, cognitive therapy, delirium monitoring, information dissemination, and patient diaries appear to be useful (10, 38–40). Among these approaches, patient diaries have shown limited evidence of the reduction of anxiety and depression (38, 39). Physical rehabilitation programs and interventions incorporating both physical and mental components are associated with a limited enhancement of the mental domain of HRQoL but more evidence is needed (38, 39). Effective patient centered interventions should be implemented as early as possible and sustained beyond hospitalization to minimize both short-term and long-term impacts of PICS on HRQoL (15, 38, 40).

The primary strength of this study lies in the inclusion of a large number of patients from seven hospitals. Validated instruments were employed, thereby enabling a reliable comparison among patients with varying PICS symptoms.

Limitations also need to be addressed. A large portion of eligible patients did not participate. Also, nonresponders more often lived alone or in a nursing home, were more fatigued, and had more symptoms of anxiety and depression at baseline. This could have led to an underestimation of results because reasons for not participating may be related to a greater perceived negative impact of an ICU stay and because these variables are negatively associated with HRQoL (29, 41–43).

Our research sample largely consisted of planned surgical patients. With these patients, the associations between ICS domains and changes in HRQoL are less apparent and probably led to an underestimation of our study results. Furthermore, we used the EQ5D-5L to assess HRQoL. Qualitative investigations revealed that several domains of HRQoL of individuals with mental disorders are not encompassed within this measurement. Had a measuring instrument been employed that encompassed these domains, associations of the mental PICS domain might have been even more pronounced (37). Also, in our analyses, we included composite scores of PICS domains instead of including health problems separately (e.g., fatigue, anxiety, etc.). This has potentially led to an overestimation of deficits and may limit the pooling of findings in future research. In addition, we used the CFQ, a subjective self-reported measurement. However, correlations between objective and subjective cognition measurements are low and therefore outcomes should be interpreted with caution (44, 45).

## CONCLUSIONS

We demonstrated that ICU treatment exerts a detrimental impact on the patient’s post-ICU HRQoL, especially when patients experienced physical, mental, or cognitive problems. It may therefore be important to prevent PICS thereby the negative impact on HRQoL. Additional research is required to ascertain the most effective programs for achieving this objective and should then be implemented as early as possible.

## Supplementary Material


