# Supplementary material for: Associations Between Physical, Cognitive, and Mental Health Domains of Post-Intensive Care Syndrome and Quality of Life: A Longitudinal Multicenter Cohort Study
Source: Crit Care Med. 2024 Oct 24;53(1):e74–86. doi: 10.1097/CCM.0000000000006461 (PMC11698138; doi:10.1097/CCM.0000000000006461)
Supplement: Supplementary file 1 [file ccm-53-e74-s001.docx]

**Supplemental file**

Supplemental table 1: Respondent’s baseline characteristics including missings

| Patient characteristics | | Respondents (N=4092) |
| --- | --- | --- |
| Age in years, n (%) | 16 – 39  40 – 64  65 -79  >80 | 253 (6.2)  1574 (38.5)  1904 (46.5)  269 (6.6) |
| Sex, n (%) | Male  Female | 2640 (64.5)  1450 (35.4) |
| Household composition, n (%) | Living alone  Living with someone else  Living in nursing home | 700 (17.1)  3338 (81.6)  54 (1.3) |
| Level of education, n (%) | Low  Medium  High | 1366 (33.4)  1715 (41.9)  948 (23.2) |
| Admission type, n (%) | Medical  Planned Surgical  Acute surgical | 1327 (32.4)  471 (11.5)  2197 (53.7) |
| Admission reason, n (%) | Cardiovascular  Respiratory  Neurologic  Gastro-intestinal  Trauma  Other | 2397 (59)  573 (14)  366 (9)  285 (7)  198 (5)  271 (6) |
| Mean APACHE IV-score (SD) |  | 54.9 (21.9) |
| Median length of ICU stay in days (IQR) |  | 1.1 (0.9-2.8) |
| >24 hours of mechanical  ventilation (%) | No  Yes | 1398 (34.2)  2602 (63.6) |
| Mean quality of life; EQ5D (SD) |  | 0.70 (0.3) |
| Fatigue; CIS, n (%) | No fatigue  Fatigue | 1425 (34.8)  2626 (64.2) |
| Cognition; CFQ, n (%) | No cognitive problems  Cognitive problems | 3592 (87.80  251 (6.1) |
| Anxiety; HADS-A, n (%) | No symptoms of anxiety  Symptoms of anxiety | 2978 (72.8)  1092 (26.7) |
| Depression; HADS-D, n (%) | No symptoms of depression  Symptoms of depression | 3023 (73.9)  1049 (25.6) |

*ICU: intensive care unit; EQ5D: European Quality of Life 5 Dimensions 5 Level Version; CIS: Checklist Individual Strength; CFQ: Cognitive Failure Questionnaire; HADS-A: Hospital Anxiety and Depression Scale-Anxiety; HADS-D: Hospital Anxiety and Depression Scale-Depression*

Supplemental table 2: Planned surgical patients’ baseline characteristics

| Patient characteristics | | Respondents (N=2197) |
| --- | --- | --- |
| Age in years, n (%) | 16 – 39  40 – 64  65 -79  >80  Missing | 69 (3.1)  842 (38.3)  1128 (51.3)  258 (7.2)  0 (0.0) |
| Sex, n (%) | Male  Female  Missing | 1525 (69.4)  671 (30.5)  1 (0.0) |
| Household composition, n (%) | Living alone  Living with someone else  Living in nursing home  Missing | 355 (16.2)  1826 (83.1)  16 (.7)  0 (0.0) |
| Level of education, n (%) | Low  Medium  High  Missing | 701 (31.9)  908 (41.3)  554 (25.2)  34 (1.5) |
| Mean APACHE IV-score (SD)  Missing, n (%) |  | 49.9 (15.1)  0 (0.0) |
| Median length of ICU stay in days (IQR)  Missing, n (%) |  | 0.96 (0.8-1.8)  0 (0.0) |
| >24 hours of mechanical  ventilation (%) | No  Yes  Missing | 620 (28.2)  1577 (71.8)  0 (0.0) |
| Mean quality of life; EQ5D (SD)  Missing, n (%) |  | 0.74 (0.2)  40 (1.8) |
| Fatigue; CIS, n (%) | No fatigue  Fatigue  Missing | 724 (33.0)  1450 (66.0)  23 (1.0) |
| Cognition; CFQ, n (%) | No cognitive problems  Cognitive problems  Missing | 1941 (88.4)  128 (5.8)  128 (5.8) |
| Anxiety; HADS-A, n (%) | No symptoms of anxiety  Symptoms of anxiety  Missing | 1569 (71.4)  618 (28.1)  10 (0.5) |
| Depression; HADS-D, n (%) | No symptoms of depression  Symptoms of depression  Missing | 1628 (74.1)  560 (25.5)  9 (0.4) |

*ICU: intensive care unit; EQ5D: European Quality of Life 5 Dimensions 5 Level Version; CIS: Checklist Individual Strength; CFQ: Cognitive Failure Questionnaire; HADS-A: Hospital Anxiety and Depression Scale-Anxiety; HADS-D: Hospital Anxiety and Depression Scale-Depression*

Supplemental table 3: Differences in baseline characteristics between responders and non-responders at 3 and 12 months follow-up

| Patient characteristics | 3 month responders (N=3368) | 3 month non-responders (n=724) | P-value | 12 month responders (n=2950) | 12 month non-responders (n=1142) | P-value |
| --- | --- | --- | --- | --- | --- | --- |
| Age, n (%)  16 – 39  40 - 64  65 -79  ->80  Missing | 192 (5.7)  1289 (38.3)  1613 (47.9)  205 (6.1)*  69 (2.0) | 61 (8.4)  285 (39.4)  291 (40.2)  64 (8.8)  23 (3.2) | <.001# | 150 (5.1)  1138 (38.6)  1428 (48.4)  170 (5.8)  64 (2.2) | 103 (9.0)*  436 (38.2)  476 (41.7)  99 (8.7)*  28 (2.5) | <.001# |
| Gender, n (%)  Male  Female  Missing | 2203 (65.4)  1163 (34.5)  2 (.1) | 437 (60.4)  287 (39.6)  0 (.0) | .009# | 1954 (66.2)  995 (33.7)  1 (.0) | 686 (60.1)  455 (39.8)  1 (.1) | <.001# |
| Household composition, n (%)  Living alone  Living with someone else  Living in nursing home  Missing | 545 (16.2)  2786 (82.7)  37 (1.1)  0 (.0) | 155 (21.4)*  552 (76.2)  17 (2.3)  0 (.0) | <.001# | 479 (16.2)  2445 (82.9)  26 (.9)*  0 (.0) | 221 (19.4)  893 (78.2)  28 (2.5)  0 (.0) | <.001# |
| Level of education, n (%)  Low  Medium  High  Missing | 1061 (31.5)  1441 (42.8)  817 (24.3)  49 (1.5) | 305 (42.1)*  274 (37.8)  131 (18.1)*  14 (1.9) | <.001# | 893 (30.3)*  1249 (42.3)  761 (25.8)*  47 (1.6) | 473 (41.4)*  466 (40.8)  187 (16.4)*  16 (1.4) | <.001# |
| Admission type, n (%)  Medical  Planned Surgical  Acute surgical  Missing | 1052 (31.2)  386 (11.5)  1856 (55.1)  74 (2.2) | 275 (38.0)*  85 (11.7)  341 (47.1)  23 (3.2) | <.001# | 894 (30.3)  341 (11.6)  1648 (55.9)  67 (2.3) | 433 (37.9)*  130 (11.4)  549 (48.1)  30 (2.6) | <.001# |
| Mean APACHE IV score (SD)  Missing, n (%) | 54.7 (21.4)  69 (2.1) | 55.8 (24.3)  23 (3.2) | .281¥ | 54.6 (20.8)  64 (2.2) | 55.8 (24.6)  28 (2.5) | .110¥ |
| Median length of ICU stay in days (IQR)  Missing, n (%) | 1.2 (0.9-2.7)  69 (2.1) | 1.5 (0.9-3.7)  23 (3.2) | .002¶ | 1.1 (0.9-2.7)  64 (2.2) | 1.5 (0.9-3.3)  28 (2.5) | .001¶ |
| Mean quality of life; EQ5D (SD)  Missing, n (%) | 0.72 (0.3)  57 (1.6) | 0.65 (0.3)  28 (3.9) | <.001¥ | 0.72 (0.3)  47 (1.6) | 0.65 (0.3)  38 (3.3) | <.001¥ |
| Fatigue; CIS, n (%)  No fatigue  Fatigue  Missing | 1222 (36.3)  2118 (63.4)  28 (.8) | 203 (28.0)  508 (70.2)  13 (1.8) | <.001# | 1090 (36.9)  1840 (62.4)  20 (.7) | 335 (29.3)  786 (68.8)  21 (1.8) | <.001# |
| Cognition; CFQ, n (%)  No cognitive problems  Cognitive problems  Missing | 2995 (88.9)  197 (5.8)  176 (5.2) | 97 (82.5)  54 (7.5)  73 (10.1) | .046# | 2640 (89.5)  164 (5.6)  146 (4.9) | 952 (83.4)  87 (7.6)  103 (9.0) | .005# |
| Anxiety; HADS-A, n (%)  No symptoms of anxiety  Symptoms of anxiety  Missing | 2518 (74.8)  838 (24.9)  12 (.4) | 460 (63.5)  254 (35.1)  10 (1.4) | <.001# | 2233 (75.7)  709 (24.0)  8 (.3) | 745 (65.2)  383 (33.5)  14 (1.2) | <.001# |
| Depression; HADS-D, n (%)  No symptoms of depression  Symptoms of depression  Missing | 2557 (75.9)  801 (23.8)  10 (.3) | 466 (64.4)  248 (34.3)  10 (1.4) | <.001# | 2284 (77.4)  659 (22.3)  7 (.2) | 739 (64.7)  390 (34.2)  13 (1.1) | <.001# |

*ICU: intensive care unit; EQ5D: European Quality of Life 5 Dimensions 5 Level Version; CIS: Checklist Individual Strength; CFQ: Cognitive Failure Questionnaire; HADS-A: Hospital Anxiety and Depression Scale-Anxiety; HADS-D: Hospital Anxiety and Depression Scale-Depression,* # significance between groups determined with Chi-square test, ¥ significance between groups determined with Independent sample t-test,¶ significance between groups determined with Mann-Whitney U test

* significance according to Bonferroni post-hoc test

Supplemental table 4: Unadjusted associations between Physical, Cognitive, and Mental PICS domains and differences in baseline and 3 months post ICU health related quality of life

| PICS domains | ß (95% CI) | Std Error | ß | P | Tolerance | VIF |
| --- | --- | --- | --- | --- | --- | --- |
| Constant | .94 (.08, .11) | .01 |  | <.001 |  |  |
| Physical PICS | -.04 (-.06, -.01) | .01 | -.06 | <.002 | .87 | 1.2 |
| Cognitive PICS | -.05 (-.09, -.02) | .01 | -.06 | <.001 | .91 | 1.1 |
| Mental PICS | -.07 (-.09, -.05) | .01 | -.12 | <.001 | .82 | 1.2 |

*PICS = post-intensive care syndrome**; QoL = quality of life; ICU = intensive care unit*

*R = .177; R2 = .031; P = <.001*

Supplemental table 5: Unadjusted associations of physical PICS and differences

between baseline and 3 months post-ICU health related quality of life

| Patient Characteristics | ß (95% CI) | Std. Error | Standardized  Coefficients ß | P |
| --- | --- | --- | --- | --- |
| Constant | .090 (.072, .109) | .009 |  | <.001 |
| Physical PICS | -.079 (-.101, .057) | .011 | -.123 | <.001 |

*PICS = post-intensive care syndrome*

*R = .123; R2 = .015; P = <.001*

Supplemental table 6: Adjusted associations of physical PICS and with differences between baseline and 3 months post-ICU health related quality of life

| Patient characteristics | ß (95% CI) | Std Error | Standardized  Coefficients ß | P | Tolerance | VIF |
| --- | --- | --- | --- | --- | --- | --- |
| Constant | .073 (.005, .142) | .035 |  | .036 |  |  |
| Physical PICS | -.076 (-.099, -.053) | .012 | -.120 | <.001 | .943 | 1.060 |
| Gender | .026 (.005, .048) | .011 | .043 | .017 | .939 | 1.065 |
| Age  • 18-39 (reference)  • 40-64  • 65-79  • >80 | -.027 (-.073, .019)  -.018 (-.064, .029)  -.014 (-.074, .046) | .023  .024  .031 | -.046  -.031  -.012 | .244  .457  .652 | .196  .180  .469 | 5.090  5.566  2.133 |
| Education  • Low (reference)  • Medium  • High | .005 (-.018, .029)  -.032 (-.059, .005) | .012  .014 | .009  -.048 | .659  .020 | .727  .717 | 1.376  1.395 |
| Admission type  • Medical (reference)  • Acute surgical  • Planned surgical | -.057 (-.091, .023)  .009 (-.015, .033) | .017  .012 | -.063  .015 | .001  .475 | .822  .697 | 1.217  1.435 |
| Household composition  • Alone (reference)  • With someone else  • Nursing home | .  023 (-.004, .051)  -.012 (-.114, .089) | .014  .052 | .031  -.004 | .095  .811 | .917  .939 | 1.091  1.065 |
| APACHE IV score | 0.00 (-.001, .000) | .000 | -.005 | .808 | .757 | 1.321 |
| Length of ICU stay | -.003 (-.005, .001) | .001 | -.068 | <.001 | .828 | 1.208 |

*PICS = post-intensive care syndrome; QoL = quality of life; ICU = intensive care unit*

*R = .180; R2 = .031; P = <.001*

Supplemental table 7: Unadjusted associations of cognitive PICS and differences

between baseline and 3 months post-ICU health related quality of life

| Patient Characteristics | ß (95% CI) | Std. Error | Standardized  Coefficients ß | P |
| --- | --- | --- | --- | --- |
| Constant | .051 | .005 |  | <.001 |
| Cognitive PICS | -.094 | .016 | -.104 | <.001 |

*PICS = post-intensive care syndrome*

*R = .104; R2 = .011; P = <.001*

Supplemental table 8: Adjusted associations of cognitive PICS and with differences between baseline and 3 months post-ICU health related quality of life

| Patient characteristics | ß (95% CI) | Std Error | Standardized  Coefficients ß | P | Tolerance | VIF |
| --- | --- | --- | --- | --- | --- | --- |
| Constant | .035 (-.032, .101) | .034 |  | .304 |  |  |
| Cognitive PICS | -.094 (-.126, -.062) | .016 | -.104 | <.001 | .977 | 1.024 |
| Gender | .023 (.002, .044) | .011 | .040 | .031 | .950 | 1.052 |
| Age  • 18-39 (reference)  • 40-64  • 65-79  • >80 | -.032 (-.078, .013)  -.021 (-.067, 025)  -.015 (-.075, .045) | .023  .024  .030 | -.057  -.038  -.013 | .158  .373  .621 | .196  .178  .465 | 5.114  5.603  2.149 |
| Education  • Low (reference)  • Medium  • High | .005 (-.018, .028)  -.025 (-.052, .001) | .012  .014 | .009  -.039 | .672  .064 | .727  .722 | 1.376  1.385 |
| Admission type  • Medical (reference)  • Acute surgical  • Planned surgical | -.051 (-.084, -.017)  .002 (-.022, .026) | .017  .012 | -.058  .004 | .004  .864 | .822  .698 | 1.217  1.433 |
| Household composition  • Alone (reference)  • With someone else  • Nursing home | .025 (-.002, .052)  .026 (-.078, .130) | .014  .053 | .033  .009 | .073  .624 | .920  .943 | 1.087  1.061 |
| APACHE IV score | 0.00 (.000, .001) | .000 | .004 | .844 | .757 | 1.321 |
| Length of ICU stay | -.003 (-.005, -.001) | .001 | -.055 | .005 | .820 | 1.220 |

*PICS = post-intensive care syndrome; QoL = quality of life; ICU = intensive care unit*

*R = .152; R2 = .023; P = <.001*

Supplemental table 9: Unadjusted associations of mental PICS and differences

between baseline and 3 months post-ICU health related quality of life

| Patient Characteristics | ß (95% CI) | Std. Error | Standardized  Coefficients ß | P |
| --- | --- | --- | --- | --- |
| Constant | .069 (.057, .081) | .006 |  | <.001 |
| Mental PICS | -.107 (-.128, -.086) | .011 | -.175 | <.001 |

*PICS = post-intensive care syndrome*

*R = .175; R2 = .031; P = <.001*

Supplemental table 10: Adjusted associations of mental PICS and with differences between baseline and 3 months post-ICU health related quality of life

| Patient characteristics | ß (95% CI) | Std Error | Standardized  Coefficients ß | P | Tolerance | VIF |
| --- | --- | --- | --- | --- | --- | --- |
| Constant | .083 (.015, .150) | .034 |  | .016 |  |  |
| Mental PICS | -.110 (-.131, -088) | .011 | -.180 | <.001 | .937 | 1.068 |
| Gender | .026 (.005, .047) | .011 | .043 | .016 | .947 | 1.056 |
| Age  • 18-39 (reference)  • 40-64  • 65-79  • >80 | -.033 (-.078, .012)  -.020 (-.066, .026)  -.017 (-.077, .042) | .023  .024  .030 | -.056  -.035  -.015 | .156  .393  .567 | .196  .179  .462 | 5.092  5.577  2.163 |
| Education  • Low (reference)  • Medium  • High | -.004 (-.028, .019)  -.040 (-.067, -.013) | .012  .014 | -.008  -.061 | .712  .003 | .721  .713 | 1.388  1.403 |
| Admission type  • Medical (reference)  • Acute surgical  • Planned surgical | -.051 (-.085, -.017)  .000 (-.023, .024) | .017  .012 | -.057  .001 | .003  .967 | .818  .694 | 1.222  1.442 |
| Household composition  • Alone (reference)  • With someone else  • Nursing home | .017 (-.011, .044)  -.016 (-.116, .084) | .014  .051 | .022  -.006 | .232  .759 | .913  .939 | 1.096  1.064 |
| APACHE IV score | .000 (-.001, .000) | .000 | -.018 | .378 | .757 | 1.321 |
| Length of ICU stay | -.003 (-.005, -.001) | .001 | -.063 | .001 | .830 | 1.204 |

*PICS = post-intensive care syndrome; QoL = quality of life; ICU = intensive care unit*

*R = .220; R2 = .048; P = <.001*

|  |
| --- |

Supplemental table 11: Adjusted associations between Physical, Cognitive, and Mental PICS domains with health related quality of life 3 months post ICU of elective surgical patients

| Patient characteristics | B | Std. Error | ß | P | tolerance | VIF |
| --- | --- | --- | --- | --- | --- | --- |
| Constant | .072 (-.009, .154) | .042 |  | .082 |  |  |
| Physical PICS | -.033 (-.056, -.010 | .012 | -.071 | .005 | .85 | 1.18 |
| Cognitive PICS | -.010 (-.048, .028) | .019 | -.013 | .594 | .91 | 1.10 |
| Mental PICS | -.061 (-.086, -.036) | .013 | -.123 | <.001 | .82 | 1.23 |
| Gender (male =1; female =2) | .024 (.001, .047) | .011 | .050 | .037 | .94 | 1.06 |
| Age |  |  |  |  |  |  |
| - 18-39 (reference) |  |  |  |  |  |  |
| - 40-64 | -.036 (-.101, .028) | .033 | -.081 | .272 | .10 | 9.8 |
| - 65-79 | -.036 (-.102, .029 ) | .034 | -.084 | .279 | .09 | 9.78 |
| - >80 | -.023 (-.099, .053) | .039 | -.026 | .552 | .29 | 3.41 |
| Education |  |  |  |  |  |  |
| - Low (reference) |  |  |  |  |  |  |
| - Medium | .014 (-.010, .038) | .012 | .032 | .256 | .69 | 1.44 |
| - High | -.017 (-.044, .010) | .014 | -.035 | .220 | .69 | 1.45 |
| Household composition |  |  |  |  |  |  |
| - Alone (reference) |  |  |  |  |  |  |
| - With someone else | .019 (-.010, .048) | .015 | .031 | .209 | .92 | 1.08 |
| - Nursing home | .254 (.110, .398) | .073 | .084 | <.001 | .95 | 1.06 |
| APACHE IV score | .000 (-.001, .001) | .000 | .019 | .491 | .76 | 1.32 |
| Length of ICU stay | -.009 (-.014, -.003) | .003 | -.077 | .002 | .91 | 1.09 |

*PICS = post-intensive care syndrome; QoL = quality of life; ICU = intensive care unit*

*R = .218; R2 = .048; P = <.001*

Supplemental table 12: Unadjusted associations between Physical. Cognitive. and Mental PICS domains and differences in baseline and 3 months post ICU health related quality of life of elective surgical patients

| PICS domains | B (95% CI) | SE B | ß | P | tolerance | VIF |
| --- | --- | --- | --- | --- | --- | --- |
| Constant | .088 (.07, .11) | .01 |  | <.001 |  |  |
| Physical PICS | -.033 (-.06, -.01) | .01 | -.07 | .005 | .89 | 1.1 |
| Cognitive PICS | .006 (-.04, .03) | .03 | .01 | .78 | .91 | 1.1 |
| Mental PICS | -.060 (-.09, -.04 | .01 | -.12 | <.001 | .83 | 1.2 |

*PICS = post-intensive care syndrome; QoL = quality of life; ICU = intensive care unit*

*R = .160; R2 = .026; P = <.001*

Supplemental table 13: Unadjusted associations between Physical, Cognitive, and Mental PICS domains and differences in baseline and 12 months post ICU health related quality of life

| PICS domains | ß (95% CI) | Std Error | ß | P | Tolerance | VIF |
| --- | --- | --- | --- | --- | --- | --- |
| Constant | .12 (.10, .14) | .01 |  | <.001 |  |  |
| Physical PICS | -.05 (-.08, -.03) | .01 | -.09 | <.001 | .82 | 1.2 |
| Cognitive PICS | -.04 (-.07, -.01) | .02 | -.05 | <.029 | .87 | 1.2 |
| Mental PICS | -.05 (-.09, -.02) | .01 | -.09 | <.001 | .77 | 1.3 |

*PICS = post-intensive care syndrome; QoL = quality of life; ICU = intensive care unit*

*R = .176; R2 = .031; P = <.001*

Supplemental table 14: Unadjusted associations of physical PICS and differences

between baseline and 12 months post-ICU health related quality of life

| Patient Characteristics | ß (95% CI) | Std. Error | Standardized  Coefficients ß | P |
| --- | --- | --- | --- | --- |
| Constant | .116 (.098, .134) | .009 |  | <.001 |
| Physical PICS | -.083 (-.105, .061) | .011 | -.143 | <.001 |

*PICS = post-intensive care syndrome*

*R = .143; R2 = .020; P = <.001*

Supplemental table 15: Adjusted associations of physical PICS and with differences between baseline and 12 months post-ICU health related quality of life

| Patient characteristics | ß (95% CI) | Std Error | Standardized  Coefficients ß | P | Tolerance | VIF |
| --- | --- | --- | --- | --- | --- | --- |
| Constant | .069 (-.004, .143) | .037 |  | .063 |  |  |
| Physical PICS | -.089 (-.111, -.066 | .012 | -.153 | <.001 | .933 | 1.071 |
| Gender | .043 (.020, .065 | .012 | .075 | <.001 | .936 | 1.069 |
| Age  • 18-39 (reference)  • 40-64  • 65-79  • >80 | -.030 (-.079, .019)  -.037 (-.088, .013)  -.042 (-.106, .023) | .025  .026  .033 | -.054  -.069  -.036 | .232  .148  .206 | .186  .169  .463 | 5.384  5.909  2.162 |
| Education  • Low (reference)  • Medium  • High | .002 (-.022, .027)  -.021 (-.049, .007) | .013  .014 | .004  -.034 | .847  .143 | .713  .696 | 1.403  1.436 |
| Admission type  • Medical (reference)  • Acute surgical  • Planned surgical | -.035 (-.070, .001)  .009 (-.016, .035) | .018  .013 | -.042  .017 | .054  .467 | .811  .683 | 1.233  1.464 |
| Household composition  • Alone (reference)  • With someone else  • Nursing home | .044 (.015, .073)  -.052 (-.170, .066) | .015  .060 | .061  -.017 | .003  .386 | .926  .949 | 1.080  1.054 |
| APACHE IV score | .000 (-.001, .000) | .000 | -.011 | .614 | .755 | 1.324 |
| Length of ICU stay | .000 (-.002, .002) | .001 | -.008 | .715 | .813 | 1.230 |

*PICS = post-intensive care syndrome; QoL = quality of life; ICU = intensive care unit*

*R = .192; R2 = .037; P = <.001*

Supplemental table 16: Unadjusted associations of cognitive PICS and differences

between baseline and 12 months post-ICU health related quality of life

| Patient Characteristics | ß (95% CI) | Std. Error | Standardized  Coefficients ß | P |
| --- | --- | --- | --- | --- |
| Constant | .073 (.063, .084) | .005 |  | <.001 |
| Cognitive PICS | -.076 (-.106, -045) | .015 | -.092 | <.001 |

*PICS = post-intensive care syndrome*

*R = .092; R2 = .008; P = <.001*

Supplemental table 17: Crude associations of cognitive PICS and with differences between baseline and 12 months post-ICU health related quality of life

| Patient characteristics | ß (95% CI) | Std Error | Standardized  Coefficients ß | P | Tolerance | VIF |
| --- | --- | --- | --- | --- | --- | --- |
| Constant | .055 (-.015, .125) | .036 |  | .126 |  |  |
| Cognitive PICS | -.079 (-.110, -.048) | .016 | -.097 | <.001 | .969 | 1.032 |
| Gender | .033 (.011, .054) | .011 | .057 | .003 | .944 | 1.059 |
| Age  • 18-39 (reference)  • 40-64  • 65-79  • >80 | -.046 (-.094, .002)  -.057 (-.106, -.007)  -.062 (-.126, .001) | .024  .025  .032 | -.084  -.105  -.054 | .061  .024  .055 | .181  .166  .463 | 5.538  6.033  2.158 |
| Education  • Low (reference)  • Medium  • High | .001 (-.023, .025)  -.018 (-.045, .009) | .012  .014 | .002  -.030 | .946  .186 | .707  .697 | 1.414  1.434 |
| Admission type  • Medical (reference)  • Acute surgical  • Planned surgical | -.035 (-.070, -.001)  .009 (-.015, .034) | .018  .012 | -.043  .017 | .043  .459 | .809  .687 | 1.236  1.455 |
| Household composition  • Alone (reference)  • With someone else  • Nursing home | .045 (.018, .073)  -.048 (-.165, .069) | .014  .060 | .063  -.016 | .001  .423 | .930  .952 | 1.075  1.051 |
| APACHE IV score | .000 (-.001, .000) | .000 | -.011 | .606 | .762 | 1.312 |
| Length of ICU stay | -.001 (-.003, .001) | .001 | -.016 | .450 | .814 | 1.229 |

*PICS = post-intensive care syndrome; QoL = quality of life; ICU = intensive care unit*

*R = .151; R2 = .023; P = <.001*

Supplemental table 18: Adjusted associations of mental PICS and differences

between baseline and 12 months post-ICU health related quality of life

| Patient Characteristics | ß (95% CI) | Std. Error | Standardized  Coefficients ß | P |
| --- | --- | --- | --- | --- |
| Constant | .090 (.078, .102) | .006 |  | <.001 |
| Mental PICS | -.085 (-.106, -.064) | .011 | -.146 | <.001 |

*PICS = post-intensive care syndrome*

*R = .146; R2 = .021; P = <.001*

Supplemental table 19: Crude associations of mental PICS and with differences between baseline and 12 months post-ICU health related quality of life

| Patient characteristics | ß (95% CI) | Std Error | Standardized  Coefficients ß | P | Tolerance | VIF |
| --- | --- | --- | --- | --- | --- | --- |
| Constant | .079 (.009, .149) | .036 |  | .027 |  |  |
| Mental PICS | -.090 (-.112, -.068 | .011 | -.154 | <.001 | .945 | 1.058 |
| Gender | .036 (.014, .057 | .011 | .063 | .001 | .947 | 1.056 |
| Age  • 18-39 (reference)  • 40-64  • 65-79  • >80 | -.045 (-.093, .002)  -.053 (-.101, -.004)  -.058 (-.120, .005) | .024  .025  .032 | -.083  -.098  -.050 | .060  .033  .071 | .184  .168  .466 | 5.448  5.935  2.145 |
| Education  • Low (reference)  • Medium  • High | -.008 (-.032, .016)  -.030 (-.057, -.003) | .012  .014 | -.014  -.049 | .523  .031 | .698  .686 | 1.433  1.458 |
| Admission type  • Medical (reference)  • Acute surgical  • Planned surgical | -.039 (-.073, -.005)  .007 (-.017, .031) | .017  .012 | -.048  .013 | .023  .581 | .809  .686 | 1.236  1.458 |
| Household composition  • Alone (reference)  • With someone else  • Nursing home | .042 (.014, .069)  -.056 (-.170, .059) | .014  .058 | .058  -.018 | .003  .340 | .926  .950 | 1.080  1.053 |
| APACHE IV score | .000 (-.001, .000) | .000 | -.013 | .540 | .762 | 1.312 |
| Length of ICU stay | -.001 (-.003, .001 | .001 | -.014 | .504 | .812 | 1.231 |

*PICS = post-intensive care syndrome; QoL = quality of life; ICU = intensive care unit*

*R = .194; R2 = .038; P = <.001*

Supplemental table 20: Adjusted associations between Physical, Cognitive, and Mental PICS domains with health related quality of life 12 months post ICU of elective surgical patients

| Patient characteristics | B | Std. Error | ß | P | Tolerance | VIF |
| --- | --- | --- | --- | --- | --- | --- |
| Constant | .100 (.005, .194) | .048 |  | .039 |  |  |
| Physical PICS | -.062 (-.088, -.037) | .013 | -.139 | <.001 | .801 | 1.25 |
| Cognitive PICS | .006 (-.034, .046) | .020 | .008 | .770 | .863 | 1.16 |
| Mental PICS | -.045 (-.074, -.017) | .014 | -.093 | .002 | .746 | 1.34 |
| Gender (male =1; female =2) | .045 (.020, .070) | .013 | .094 | <.001 | .942 | 1.06 |
| Age |  |  |  |  |  |  |
| - 18-39 (reference) |  |  |  |  |  |  |
| - 40-64 | -.064 (-.141, .013) | .039 | -.144 | .101 | .087 | 8.49 |
| - 65-79 | -.081 (-.160, -.003) | .040 | -.187 | .041 | .080 | 8.53 |
| - >80 | -.100 (-.189, -.012) | .045 | -.113 | .026 | .259 | 3.86 |
| Education |  |  |  |  |  |  |
| - Low (reference) |  |  |  |  |  |  |
| - Medium | .012 (-.015, .039) | .014 | .027 | .389 | .678 | 1.48 |
| - High | .005 (-.025, .035) | .015 | .010 | .742 | .666 | 1.50 |
| Household composition |  |  |  |  |  |  |
| - Alone (reference) |  |  |  |  |  |  |
| - With someone else | .030 (-.002, .062) | .016 | .050 | .065 | .929 | 1.08 |
| - Nursing home | .225 (.073, .377) | .078 | .078 | .004 | .936 | 1.07 |
| APACHE IV score | .000 (-.001, .001) | .000 | .014 | .640 | .780 | 1.28 |
| Length of ICU stay | -.004 (-.011, .002) | .003 | -.033 | .211 | .955 | 1.05 |

*PICS = post-intensive care syndrome; QoL = quality of life; ICU = intensive care unit*

*R = .245; R2 = .06; P = <.001*

Supplemental table 21: Unadjusted associations between Physical. Cognitive. and Mental PICS domains and differences in baseline and 12 months post ICU health related quality of life of elective surgical patients

| PICS domains | B (95% CI) | SE B | ß | P | tolerance | VIF |
| --- | --- | --- | --- | --- | --- | --- |
| Constant | .119 (.10, .14) | .01 |  | <.001 |  |  |
| Physical PICS | -.059 (-.08, -.03) | .01 | -.13 | <.001 | .82 | 1.2 |
| Cognitive PICS | .012 (-.03, .05) | .02 | .02 | .553 | .88 | 1.1 |
| Mental PICS | -.047 (-.08, -.02) | .014 | -.09 | .001 | .79 | 1.27 |

*PICS = post-intensive care syndrome; QoL = quality of life; ICU = intensive care unit*

*R = .187; R2 = .035; P = <.001*

STROBE Statement—checklist of items that should be included in reports of observational studies

|  | | **Item No** | **Recommendation** | | **Page  No** |
| --- | --- | --- | --- | --- | --- |
| **Title and abstract** | | 1 | (*a*) Indicate the study’s design with a commonly used term in the title or the abstract | | 2 |
|  |  |  | (*b*) Provide in the abstract an informative and balanced summary of what was done and what was found | | 2 |
| **Introduction** | | | | | |
| Background/rationale | | 2 | Explain the scientific background and rationale for the investigation being reported | | 4 |
| Objectives | | 3 | State specific objectives, including any prespecified hypotheses | | 4/5 |
| **Methods** | | | | | |
| Study design | | 4 | Present key elements of study design early in the paper | | 5 |
| Setting | | 5 | Describe the setting, locations, and relevant dates, including periods of recruitment, exposure, follow-up, and data collection | | 5 |
| Participants | | 6 | (*a*) *Cohort study*—Give the eligibility criteria, and the sources and methods of selection of participants. Describe methods of follow-up  *Case-control study*—Give the eligibility criteria, and the sources and methods of case ascertainment and control selection. Give the rationale for the choice of cases and controls  *Cross-sectional study*—Give the eligibility criteria, and the sources and methods of selection of participants | | 5 |
|  |  |  | (*b*) *Cohort study*—For matched studies, give matching criteria and number of exposed and unexposed  *Case-control study*—For matched studies, give matching criteria and the number of controls per case | |  |
| Variables | | 7 | Clearly define all outcomes, exposures, predictors, potential confounders, and effect modifiers. Give diagnostic criteria, if applicable | | 5-7 |
| Data sources/ measurement | | 8* | For each variable of interest, give sources of data and details of methods of assessment (measurement). Describe comparability of assessment methods if there is more than one group | | *5-7* |
| Bias | | 9 | Describe any efforts to address potential sources of bias | | 7-8 |
| Study size | | 10 | Explain how the study size was arrived at | | 8 |
| Quantitative variables | | 11 | Explain how quantitative variables were handled in the analyses. If applicable, describe which groupings were chosen and why | | 5-8 |
| Statistical methods | | 12 | (*a*) Describe all statistical methods, including those used to control for confounding | | 7-8 |
|  |  |  | (*b*) Describe any methods used to examine subgroups and interactions | | 7-8 |
|  |  |  | (*c*) Explain how missing data were addressed | | 7-8 |
|  |  |  | (*d*) *Cohort study*—If applicable, explain how loss to follow-up was addressed  *Case-control study*—If applicable, explain how matching of cases and controls was addressed  *Cross-sectional study*—If applicable, describe analytical methods taking account of sampling strategy | | n/a |
|  |  |  | (*e*) Describe any sensitivity analyses | |  |
| **Results** | | | | | |
| Participants | 13* | (a) Report numbers of individuals at each stage of study—eg numbers potentially eligible, examined for eligibility, confirmed eligible, included in the study, completing follow-up, and analysed | | 8 | |
|  |  | (b) Give reasons for non-participation at each stage | | Fig 1 | |
|  |  | (c) Consider use of a flow diagram | | Fig 1 | |
| Descriptive data | 14* | (a) Give characteristics of study participants (eg demographic, clinical, social) and information on exposures and potential confounders | | 8/9 | |
|  |  | (b) Indicate number of participants with missing data for each variable of interest | | Table 1 | |
|  |  | (c) *Cohort study*—Summarise follow-up time (eg, average and total amount) | | 8/9 | |
| Outcome data | 15* | *Cohort study*—Report numbers of outcome events or summary measures over time | | *8/9* | |
|  |  | *Case-control study—*Report numbers in each exposure category, or summary measures of exposure | | *n/a* | |
|  |  | *Cross-sectional study—*Report numbers of outcome events or summary measures | | *8/9* | |
| Main results | 16 | (*a*) Give unadjusted estimates and, if applicable, confounder-adjusted estimates and their precision (eg, 95% confidence interval). Make clear which confounders were adjusted for and why they were included | | 9/10 | |
|  |  | (*b*) Report category boundaries when continuous variables were categorized | | 6/7 | |
|  |  | (*c*) If relevant, consider translating estimates of relative risk into absolute risk for a meaningful time period | | n/a | |
| Other analyses | 17 | Report other analyses done—eg analyses of subgroups and interactions, and sensitivity analyses | | 9/10 | |
| **Discussion** | | | | | |
| Key results | 18 | Summarise key results with reference to study objectives | | 10 | |
| Limitations | 19 | Discuss limitations of the study, taking into account sources of potential bias or imprecision. Discuss both direction and magnitude of any potential bias | | 12 | |
| Interpretation | 20 | Give a cautious overall interpretation of results considering objectives, limitations, multiplicity of analyses, results from similar studies, and other relevant evidence | | 10/11 | |
| Generalisability | 21 | Discuss the generalisability (external validity) of the study results | | 10-12 | |
| **Other information** | | | | | |
| Funding | 22 | Give the source of funding and the role of the funders for the present study and, if applicable, for the original study on which the present article is based | | 13 | |
